# Impact of COVID-19 on referrals for physical and mental health care

**DOI:** 10.1192/bjo.2021.270

**Published:** 2021-06-18

**Authors:** Petros Lekkos, Joanna Thorpe, Janet Obeney-Williams, Fiona Xing

**Affiliations:** 1 Camden and Islington NHS Foundation Trust; 2 Whittington Health NHS Trust, Camden and Islington Foundation NHS Trust

## Abstract

**Aims:**

To determine the effect of the COVID-19 pandemic on referrals to mental health and physical health services.

**Method:**

We analysed referral data from three psychiatric services in the boroughs of Camden and Islington across 2018-2020: Early Intervention Services (for patients with a 1st episode of psychosis), Crisis Resolution Teams and inpatient admissions. We also analysed GP referral data to Cancer Services (two-week wait referrals) to Whittington Hospital, Royal Free Hospital and University College Hospital (all of North Central London). We examined the impact of the COVID-19 pandemic on these referrals and compared the findings between physical and mental health. We chose to use EIS and Cancer services as comparable services since they both operate with the two-week target of achieving diagnosis of psychosis and cancer respectively.

**Result:**

The number of referrals to EIS and CRT both decreased to 61% in April 2020 with respect to their baseline; EIS referrals continued to decrease to 48% in May before starting to recover. Inpatient admissions saw a smaller reduction to 87% in April 2020. The number of cancer two-week wait referrals similarly decreased and reached a trough of 37% in April 2020. The rate of recovery back to the baseline number of referrals and admissions relative to previous years differed between services, with acute care recovering faster. Referrals to CRT and inpatient admissions recovered by 98% and 115% respectively by June 2020; comparatively, referrals to EIS recovered to 102% by December 2020. In contrast, cancer two-week wait referrals returned to 106% by September 2020, a rate faster than EIS, but slower than CRT and inpatient admissions.

**Conclusion:**

The reduction in the number of referrals across all examined services correlated with the first wave of the COVID-19 pandemic. The rate of decrease was similar across all services, coinciding with the peak of COVID-19 infections. However, the ultimate degree of decrease and following rate of recovery in numbers differed across both psychiatric and non-psychiatric services. These differences likely have multifactorial origins. The authors discuss contributing factors, such as changes in health seeking behaviours observed during the pandemic, potential impact of reduction in face to face consultations in primary care, as well as temporary changes in the population demographic of Camden and Islington resulting in absent target groups (i.e. students who make up a large proportion of referrals to EIS opting to return home). It remains important to not neglect mental health and face a hidden epidemic once COVID-19 pandemic settles.

